# Reactivity of aragonite with dicalcium phosphate facilitates removal of dental calculus

**DOI:** 10.1007/s10856-025-06867-6

**Published:** 2025-03-15

**Authors:** Amir Elhadad, Tayebeh Basiri, Ashwaq Al-Hashedi, Sophia Smith, Hanan Moussa, Sadiya Veettil, Eva Mª Pérez Soriano, Faleh Tamimi

**Affiliations:** 1 Department of Pre-Clinical Oral Health Sciences, College of Dental Medicine, QU-Health, Qatar University, Doha, Qatar; 2 Faculty of Dentistry, McGill University, Montreal, QC Canada; 3 Departments of Material Engineering, McGill University, Montreal, QC Canada; 4 Department of Applied Oral Sciences, Faculty of Dentistry, Dalhousie University, Halifax, NS Canada; 5 Escuela Politécnica Superior, Universidad de Sevilla, Seville, Spain

## Abstract

Dental calculus, a main contributor of periodontal diseases, is mostly composed of inorganic calcium phosphate species such as dicalcium phosphate, whitlockite, octa calcium phosphate, and hydroxyapatite. Under physiological pH 7.4, dicalcium phosphates can gradually interact with calcium carbonate to form hydroxyapatite. Therefore, we hypothesized that aragonite (Arg) could react with dental calculus, facilitating its removal. To assess the reactivity of Arg with dental calculus, we examined the changes in surface morphology, composition, and topography of Arg and dental calculus upon exposure to each other in an aqueous environment. The impact of Arg on the removal of dental calculus was assessed by brushing polished sections of dental calculus, enamel, and dentin with slurries of Arg and measuring the depth of abrasion using a stylus profilometer. Our results demonstrate that Arg can react with dental calculus in aqueous environment. This reaction increases calculus surface roughness which in turn facilitate dental calculus removal by brushing. Aragonite could be a promising abrasive for toothpaste design for management of dental calculus.

**Graphical Abstract:**

**Figure:**
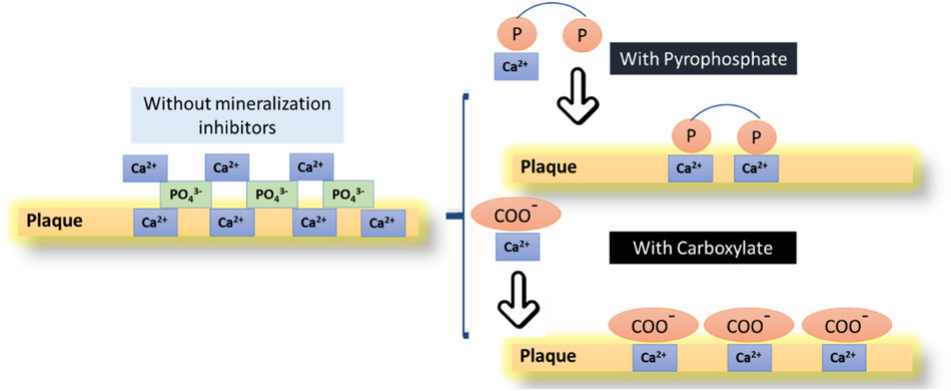
This study proposes an innovative approach for the softening and removal of dental calculus based on the use of aragonite. This novel approach, which takes advantage of the chemical reactivity between aragonite and the minerals found in dental calculus, opens the door for developing homecare products that could help patients and clinicians more effectively control and manage dental calculus deposits. Anti-calculus Action. Pyrophosphate and carboxylate inhibit calculus formation by preventing calcium phosphate deposition in plaque.

## Introduction

**Fig. 1:**
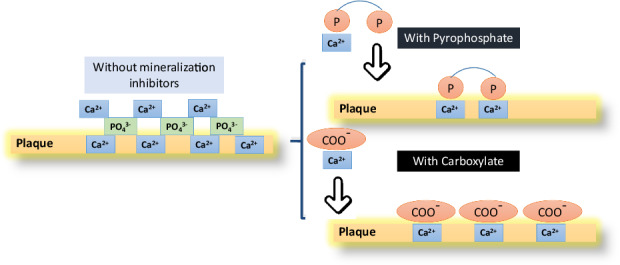
Anticalculus Action. Pyrophosphate and carboxylate inhibit calculus formation by inhibiting calcium phosphate deposition in plaque

Dental calculus is mineralized plaque that adheres to the surfaces of teeth [1, 2]. Its porous nature favors the bacteria colonization and the development of periodontitis by toxins [3, 4]. Therefore, removal and growth inhibition of dental calculus are imperative for periodontal disease prevention [5, 6]. Currently, the only feasible method for removing dental calculus is mechanical removal by scaling in dental clinics [7, 8]. Toothpastes may also include a variety of abrasives for cleaning and removal of biofilms, (i.e., calcite, silicon dioxide, brushite, and gibbsite). However, escalating abrasive levels to combat dental calculus may result in damage to enamel and dentin [9–11]. Additionally, toothpaste formulations may include mineralization inhibitors, such as carboxylates and pyrophosphates, to prevent calculus formation. Nevertheless, increasing their concentration in order to remove calculus could result in the dissolution of dentin and enamel. Calcium phosphate crystals constituting dental calculus are formed when calcium and phosphate ions react to form precipitates [12–14]. Toothpastes containing carboxylates or pyrophosphate- aid in preventing calculus formation by preventing amorphous calcium phosphate from crystallizing into hydroxyapatite [15, 16]. These agents function by dissolving the calcium in dental plaque, thereby impeding crystal formation [15, 16] or binding to the surface of growing crystals to hinder their growth (Fig. 1).

Despite their low toxicity, chelating agents such as pyrophosphates and carboxylates can prevent hydroxyapatite from crystallizing in bones and teeth and may negatively affect the equilibrium between demineralization and remineralisation at the surface of the tooth [16, 17]. Owing to these issues, alternative strategies are required to eliminate dental calculus while preserving the tooth’s underlying structure.

Approximately 15–20% of calculus comprises organic constituents like proteins, glycoproteins, lipids, DNA, carbohydrates, and microorganisms [12, 13, 18]. Nevertheless, it is mainly inorganic and primarily composed of calcium carbonate and phosphates, along with trace amounts of carbonate, sodium, magnesium, silicon, iron, and fluoride, manifested in the form of minerals such as aragonite, whitlockite, octacalcium phosphate, and hydroxyapatite [12, 13, 19]. Aragonite minerals are made of Ca^2+^ and ${{\rm{PO}}}_{4}^{3-}$ ions that can be found in both dental calculus and enamel, however aragonite also includes ${{\rm{HPO}}}_{4}^{2-}$ ions that are exclusively found in calculus. Thus, targeting ${{\rm{HPO}}}_{4}^{2-}$ removal could potentially enhance calculus removal while preserving dentin and enamel integrity, than the approach that focuses on Ca^2+^.

One method to capture HPO_4_^2^ ions involves reacting them with calcium carbonate. Calcium carbonate may exist in three distinct mineral forms: calcite is the stable form, vaterite, whereas aragonite (Arg) is metastable and can eventually change into calcite with time or heat [20]. The needle-like aragonite crystals have a larger surface area than the rhombohedral calcite crystals, which could cause them to react faster. Dicalcium phosphate is known to react with calcium carbonate (aragonite) minerals, according to the following equation [21, 22].

1 $$3{\rm{CaHP}}{{\rm{O}}}_{4}{\cdot }2{{\rm{H}}}_{2}{\rm{O}}+2{\rm{CaC}}{{\rm{O}}}_{3}\to {{{\rm{Ca}}}_{5}\left({{\rm{PO}}}_{4}\right)}_{3}{\rm{OH}}+2{{\rm{CO}}}_{2}+7{{\rm{H}}}_{2}{\rm{O}}$$

Hence, we hypothesized that the removal of the dental calculus could be facilitated by the reaction of the Arg with diphosphate ions of dental calculus. To test this hypothesis, the reactivity of aragonite with dental calculus has been investigated.

## Materials & methods

**Fig. 2:**
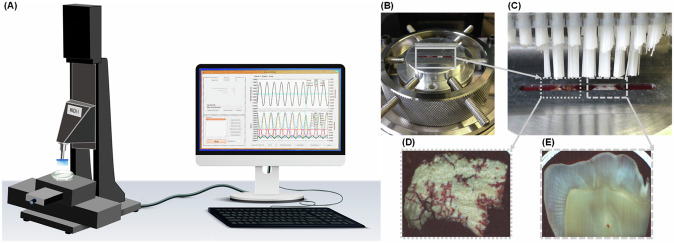
**A**, **B** Customized mechanical brushing system showing the mounted resin mold, (**C**) resin-embedded sections mounted in the Mach-1 instrument, (**D**) resin-embedded calculus sections, and (**E**) resin-embedded enamel/dentin sections

After obtaining the ethical approval from the McGill University Health Center Ethical Committee (A01-E02-18A), sections of enamel, dentin, and calculus (4 × 4 × 3 mm), (*n* = 12), were obtained from freshly extracted human teeth. These sections underwent a 10-min cleaning process in an ultrasonic distilled water bath before being embedded in acrylic resin (Fig. 2C, D). The resin-embedded sections were then meticulously polished to achieve smooth surfaces on the enamel, dentin, and calculus. This was done using successively finer silicon carbide abrasives # 600-1200 (Grit C-wt, AA abrasive Philadelphia, PA). To obtain a flawless finish, colloidal silica suspension was used as the polishing slurry (0.06 m; Master Met; Buehler, USA), along with two different types of reusable polishing cloths (15–0.02 m; TexMet C, 1–0.02 m; ChemoMet). Subsequently, the polished resin-embedded sections were then cleaned with distilled water in an ultrasonic bath for 20 min before being dried.

Two sources of aragonite were examined in this study: aragonite from phytoplankton (see Supplementary Data for oolitic aragonite) [23] and aragonite obtained from cuttlefish bone [24–26]. Aragonite (Arg) obtained from cuttlefish bones underwent a cleaning process to remove any residual flesh and then dried. The dried aragonite minerals were grounded using an ultra-centrifugal rotor mill and sieved through a sieve a mess with a cut-off between 55 and 65 µm. To enhance the reactivity of Arg, the sieved powder was treated with ammonium chloride (NH_4_Cl), at 20 °C in a solution comprising 55% water, 35% Arg powder, and 10% NH_4_Cl. The mixture was subsequently centrifuged, sieved (with an opening of 55 µm), filtered, water-washed, and finally dried in a tunnel drier.

The aragonite specimens were characterized as follows. The specific surface area (SSA) was determined using a nitrogen adsorption isotherm (TriStar 3000, Micrometrics GA, USA) at 77.3 K. Prior to analysis, the specimens underwent overnight degassing in nitrogen at 120 °C (Micrometrics Flow prep 060) to remove water from moisture. A Horiba laser particle size analyser (Model LA-920, Kyoto, Japan) was used to obtain the powder particle size distribution (PSD).

Thermal properties were assessed using thermogravimetry and differential scanning calorimetry TGA/DSC (TGA/DSC 1 from Mettler Tolledo). In an open pan (Al_2_O_3_) attached to a microbalance, 5 mg of dry powder samples were heated under nitrogen flow from 30 to 700 °C at a rate of 10 °C min^−1^. Crystallographic properties were explored with X-ray diffraction (XRD), (Bruker AXS GmbH, Karlsruhe, Germany), using a Cu K_α_ radiation source (K = 1.5406 Å), operated at 40 kV and 40 mA, within the 2ϴ range of 10 to 60° using a step scan mode with steps of 0.02° and counting time of 4 s per step. To identify functional groups, infrared (IR) spectra of the powder samples were acquired using a Bruker Tensor 27 Fourier transform infrared (FTIR) spectrometer with an accumulation of 64 scans in the range of 400–4000 cm^−1^ at a resolution of 4 cm^−1^.

### Reaction of aragonite with calcium phosphate solution

To assess the impact of TArg on calculus inhibition, we investigated its reactivity with a calcium phosphate solution. Briefly, 0.5 g of TArg was added into 200 ml of a calcium phosphate supersaturated growth solution in a covered glass beaker containing 15.0 mM CaCl_2_ and 15.0 mM Na_2_HPO_4_. The solution pH was adjusted to 7.60 ± 0.2 by stepwise addition of 1.0 M hydrochloric acid (HCl) (Sigma-Aldrich, USA) at 20 °C. A pure CaP solution was prepared and kept under identical conditions as a control. Natant samples were collected after one hour, 1, 3, 7, and 14 days by centrifuging the precipitate for 15 min at 10,000 rpm. These collected natant samples were dried, before undergoing examination of their properties using XRD and FTIR.

### Reaction of TArg with dental calculus

To assess the reactivity of the TArg with calculus, sections of dental calculus (*n* = 12) were incubated in a TArg slurry, TArg:dd-H_2_O, [1:1, (w:w)] for 1 h. Subsequently, the slurries were filtered using Whatman quantitative filter papers, followed by rinsing with dd-H_2_O and air drying. The calculus surface and aragonite were then analyzed using GA-XRD, SEM-EDX, and ATR-IR techniques. Additionally, a 3D Optical Surface Profiler (NewViewTM 8000, ZYGO, Connecticut, USA) was employed to assess changes in calculus surface roughness after exposure to aragonite slurry.

### Brushing of dental calculus

A brushing test developed in our lab was used to assess the abrasiveness of TArg-slurry on dental calculus. Polished enamel, dentin, and calculus sections were affixed to a custom-built brushing machine (Mach-1, Biomomentum, QC) (Fig. 2A, B) using a thin mask that exposed only 0.5 × 15 mm of the sample to the brush (Fig. 2D, E). A toothbrush was positioned perpendicular to the sample surface within the apparatus, aligned with the mask opening (Fig. 2C). Employing the TArg slurry, TArg:dd-H_2_O, [1:1, (w:w)], or a control toothpaste (Colgate® total toothpaste), the calculus, dentin, and enamel sections were brushed for 1 h at a speed of 90 strokes per minute (equivalent to 5400 cycles), with an applied weight of 500 g. This regimen simulates two weeks of regular, twice-daily, 2-min teeth brushing. The effectiveness of the slurries in calculus removal was evaluated by measuring the abrasion depth using a stylus profilometer (Dektak XT TM, Bruker, United States). The portion of sample surface untouched by the brush served as the baseline for assessing the abrasion depth. Statistical analysis was conducted with each sample in triplicate, and the results derived were expressed as mean values ± standard deviation. For comparing two groups, we used the paired T-test, and for three-way comparisons, we employed the Kruskal–Wallis test.

## Results

### Impact of NH_4_Cl treatment on the Aragonite (Arg)

**Fig. 3:**
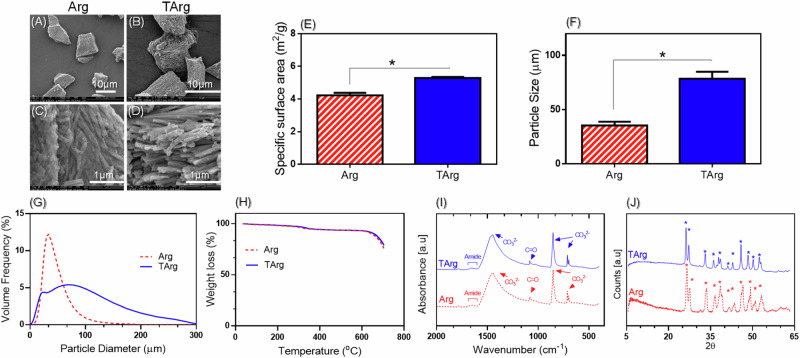
Characterization of calcite, aragonite (Arg), treated aragonite (TArg) powder: **A**, **B** SEM micrographs, (**C**), (**D**) Higher magnification, (**E**) Specific surface area (*p* < 0.05), (**F**) Particle size (*p* < 0.05), (**G**) Particle size distribution, (**H**) TGA, (**I**) FTIR, and (**J**) XRD

Using a variety of techniques, the effect of NH_4_Cl treatment on the morphology, structure, composition, and surface area of the aragonite was evaluated. SEM micrographs revealed that Arg exhibits a needle-like structure (Fig. 3A, C), which becomes more evident and well-defined after being treated with NH_4_Cl (Fig. 3B, D).

Post-treatment with NH_4_Cl, the TArg surface area and particle size increased. This augmentation in particle size may stem from particle agglomeration driven by changes in Zeta potential or the dissolution of small particles as a result of the NH_4_Cl treatment. Specifically, Zeta potentials shifted from of −17.35 ± 0.78 to −12.04 ± 0.60 (mV), respectively.

The TGA curves for the Arg and TArg specimens are depicted in Fig. 3H. The TGA curve of the Arg sample (Arg) showed weight losses at three distinct temperature ranges, 1.3% weight loss between 30 and 130 °C related to the removal of moisture, 3.8% weight loss between 130 and 355 °C attributed to the combustion of organics [27, 28], and a final weight loss between 650 and 700 °C, indicative of the decomposition of CaCO_3_ into CaO and CO_2_ [29, 30]_._ Compared to Arg, TArg’s degradation occurred at slightly higher temperatures, indicating a decrease of the organic content and an increased crystallinity in the TArg as a result of NH_4_Cl treatment [31].

FTIR spectra (Fig. 3I) of Arg and TArg showed peaks at 699, 850, and 1422 cm^−1^, signifying the presence of carbonate groups characteristic of aragonite [32, 33]. These peaks were shifted in the spectra of the TArg, appearing at 710, 870, and 1360 cm^−1^. Additionally, tiny bands at 1551 and 1317 cm^−1^ in the spectra of Arg and TArg were indicative of amide II bending and amide III stretching, respectively, implying the presence of organic traces [34].

The XRD patterns of the Arg and TArg specimens displayed peaks characteristic of aragonite as the primary crystalline phase (Fig. 3J) [35]. Notably, the TArg peaks were more defined and narrower, suggesting that NH_4_Cl treatment enhanced the Arg crystallinity [35].

### Reactivity of Aragonite with free ${{\mathbf{PO}}}_{\bf{4}}^{\bf{3-}}$ ions

**Fig. 4:**
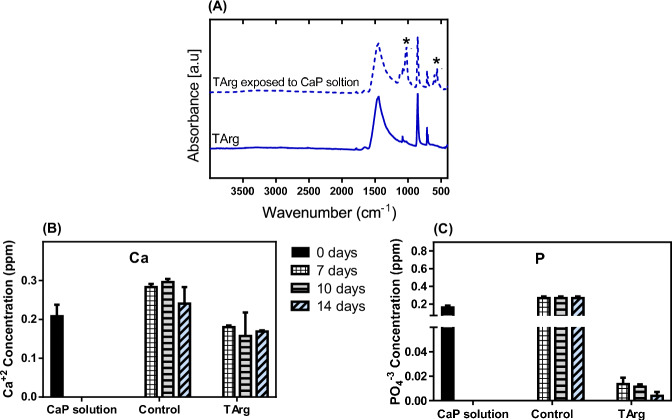
**A** FTIR spectra of TArg powder before and after 14 days of incubation in saturated CaP solution (*: ${{\rm{PO}}}_{4}^{3-}$ group), (**B**) Ca^+2^ concentration in the supernatant, and (C) ${{\rm{PO}}}_{4}^{-3}$ concentration in the supernatant

The precipitation of CaP was investigated in the presence of TArg (Fig. 4). Following 14 days of incubation in a supersaturated CaP solution, the FTIR spectra of TArg exhibited signals corresponding to ${{\rm{PO}}}_{4}^{3-}$ bands at 1035, 1023, 600, and 560 cm^−1^ (Fig. 4A). Moreover, the concentration of Ca^2+^ in the CaP solution slightly decreased (Fig. 4B), whereas the concentration of P ions^−^ decreased substantially after exposure to TArg (Fig. 4C). This suggests that TArg has the capability to react with free ${{\rm{PO}}}_{4}^{3-}$ ions and remove them from the surrounding solution, implying that TArg can function as a scavenger for free phosphate.

### Effect of dental calculus on TArg

**Fig. 5:**
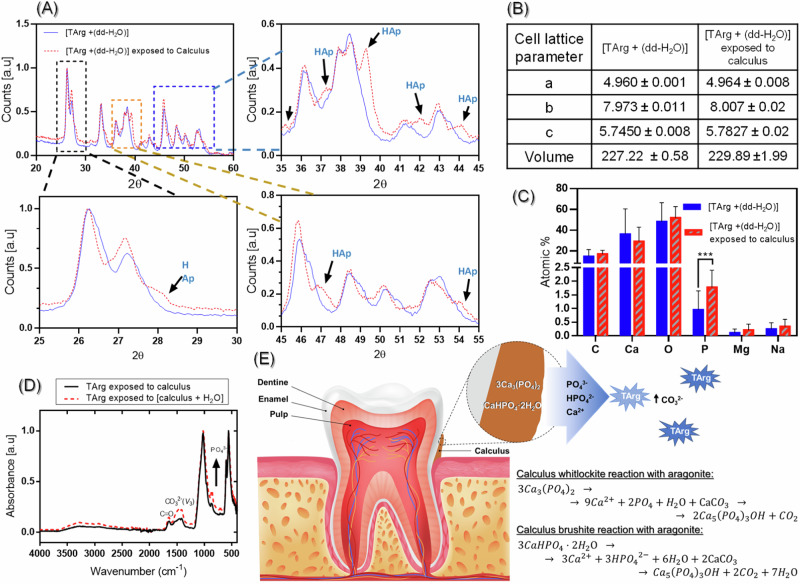
Characterization of TArg slurry before and after exposure to calculus, (**A**) XRD graphs, (**B**) crystallographic parameters, (**C**) EDX analysis (*p < 0.05, ***p < 0.001), (**D**) FTIR spectra of the TArg after exposure to calculus, and (**E**) Schematic diagram representing the reaction between aragonite (TArg) and dental calculus

Exposure to calculus induced a change in the pH of the TArg slurry form 7.92 ± 0.05 to 8.08 ± 0.02, potentially indicating an early stage of TArg reaction with calculus. XRD analysis unveiled crystallographic alterations in TArg upon contact with dental calculus (Fig. 5A), with aragonite peaks displaying a general shift towards lower angles. Additionally, new diffraction peaks emerged at 2θ 27.9, 35.2, 37.5, 39.4, 42.0, 44.1, 46.9, and 54.1° after exposure of TArg to calculus. These new peaks are hydroxyapatite-related and could indicate that TArg reacts with phosphate ions from dental calculus. EDX analysis further revealed that the elemental composition of TArg changed after exposure to dental calculus, as demonstrated by a significant rise in phosphorus concentration (Fig. 5C). Figure 5E illustrates the potential reaction between aragonite (TArg) and dental calculus. Furthermore, FTIR spectra of TArg after exposure to calculus revealed heightened absorption intensities of C = O, ${{\rm{CO}}}_{3}^{2-}$, and ${{\rm{PO}}}_{4}^{3-}$ bands, at 1636, 1420, and 850 cm^−1^ respectively, in calculus after exposure to TArg slurry. This corroborates changes in calculus composition following exposure to TArg slurry.

### Dental calculus reactivity with Aragonite

**Fig. 6:**
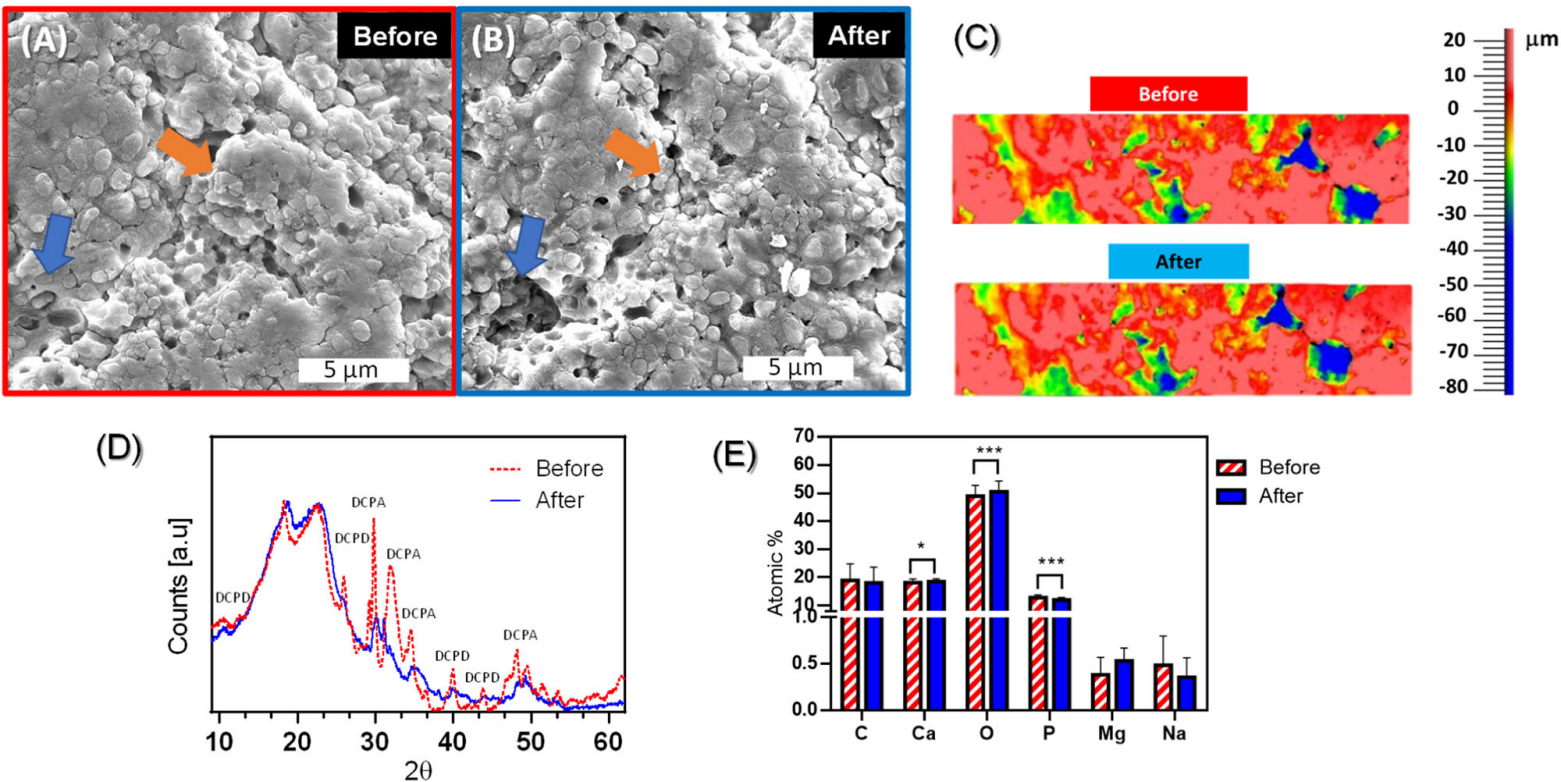
characterization of dental calculus surface before and after exposure to TArg slurry. **A**, **B** SEM micrographs of calculus before and after exposure to TArg slurry. **C** 3D optical surface profiler showing changes of surface roughness of calculus as a result of TArg exposure. **D** GA-XRD showing the changes of crystallinity of calculus after exposure to TArg slurry [DCPD: Brushite (Dicalcium phosphate dihydrate, (CaHPO_4_·2H_2_O) and DCPA: Monetite (Dibasic calcium phosphate anhydrate, CaHPO_4_)]. **E** EDX analysis showing the changes of elemental composition of calculus before and after 1 h of exposure to TArg slurry (*p < 0.05)

After being exposed to TArg slurry, the surface composition, structure, morphology, and roughness of dental calculus changed (Fig. 6). SEM showed that exposure to TArg renders the calculus surface more porous and rougher (see arrows in Fig. 6B). The arrows highlight the increased porosity and roughness in dental calculus after TArg exposure, directing attention to key structural changes. Optical surface profilometer (Fig. 6D) further confirmed the increase in calculus surface roughness because of exposure to TArg slurry. GA-XRD analysis showed that calculus crystallinity decreased after being exposed to TArg slurry, as demonstrated by a decrease in peak intensity and a relative increase in the diffraction peak width (Fig. 6E). Additionally, some crystallographic peaks that were visible in calculus before exposure to TArg corresponding to diphosphate minerals (HPO_4_^2^) [36] disappeared after exposure of calculus to TArg. Furthermore, EDX analysis of the calculus surface (Fig. 6F) revealed that phosphorus content decreased while the oxygen content increased.

### Abrasion experiments comparing TArg slurries, toothpaste with TArg, and commercially available toothpaste

**Fig. 7:**
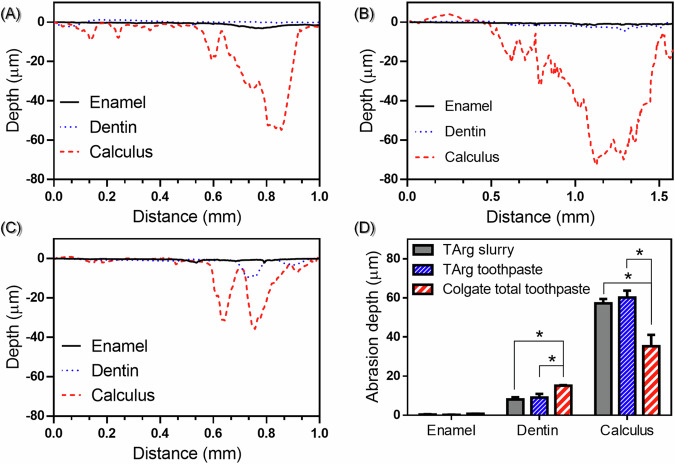
Abrasion depth of (**A**); TArg slurry, (**B**); toothpaste containing TArg, and (**C**); Colgate Total toothpaste on enamel, dentin, and calculus, (**D**); the amount that was removed by each of them (*p 0.05)

According to the brushing test on surfaces of calculus, enamel, and dentin (Fig. 7), all products were more abrasive against calculus and less abrasive against enamel. Moreover, the TArg slurries and toothpaste containing TArg were significantly less abrasive against dentin and enamel but more abrasive against dental calculus as compared to Colgate toothpaste.

## Discussion

Our experiments have demonstrated that aragonite can effectively aid in the removal of dental calculus through chemical reactions with the CaP constituents present in calculus. Specifically, we have shown that aragonite is capable of reacting with CaP species found in calculus such as brushite through ion exchange interactions, leading to the partial dissolution of the calculus surface and the subsequent precipitation of hydroxyapatite on aragonite particles.

Furthermore, these findings indicate that aragonite can also interact with free Ca and P ions, effectively removing them from the aqueous environment. This suggest that aragonite may serve as an inhibitor of mineralization and calculus formation. Indeed, clinical studies have demonstrated the efficacy of aragonite toothpaste in preventing calculus formation [37]. Additionally, besides its reactivity with free phosphate ions, this research highlights that aragonite could react with calcium phosphate species present in dental calculus, as well as with dental calculus itself.

It is worth noting that aragonite effect on calculus does not extend to enamel due to its reactivity with brushite, the metastable phase constituent of calculus, in contrast to the stable hydroxyapatite phase constituent of enamel, as reported by many researchers and supported by the FTIR results [21, 22].

Traditionally, efforts to manage calculus have centered around calcium chelation using molecules such as pyrophosphate and carboxylate [38, 39]. These molecules prevent deposition of calculus by interaction with Ca, however, they are not able to remove calculus at low concentration and, if use at high concentrations, they can lead to tooth demineralization. Our study introduces a novel strategy for dental calculus management that focuses on phosphate removal instead of calcium chelation, enabling calculus removal without damaging the tooth.

As an additional validation of the findings, oolitic aragonite (derived from Phytoplankton) was employed as an alternative source of natural aragonite (see Supplementary Data).

## Conclusion

Dental Calculus could be eliminated by removing the ${{\rm{HPO}}}_{4}^{2-}$ ions. This allows for selective abrasion of calculus without damaging the dentin and enamel. Aragonite can react with Dicalcium Phosphate in Dental calculus through ion exchange interaction.

## Supplementary information

Supplementary Material
