# Supplementary material for: Reactivity of aragonite with dicalcium phosphate facilitates removal of dental calculus
Source: J Mater Sci Mater Med. 2025 Mar 15;36(1):27. doi: 10.1007/s10856-025-06867-6 (PMC11910395; doi:10.1007/s10856-025-06867-6)
Supplement: Supplementary file 1 — Supplementary Material [file 10856_2025_6867_MOESM1_ESM.docx]

**Supplementary Material for**

**Reactivity of Aragonite with Dicalcium Phosphate Facilitates Removal of Dental Calculus**

Amir Elhadad^1^, Tayebeh Basiri^2^, Ashwaq Al-Hashedi^2^, Sophia Smith^3^, Hanan Moussa^4^, Sadiya Veettil^1^, Eva Mª Pérez Soriano^5^, Faleh Tamimi^1^

Corresponding author:

Faleh Tamimi, Ph.D.

Professor of Restorative Dentistry

College of Dental Medicine

Qatar University

E-mail: [fmarino@qu.edu.qa](mailto:fmarino@qu.edu.qa)

**Contents**

1. **Materials and Methods**
2. **Supplementary Figures**

Figure S2.1. (A-D) SEM micrographs of the phytoplankton Aragonite, (E-F) XRD and FTIR of TArg obtained from two different sources; (Black) Cuttlefish bone and phytoplankton (Red)

Figure S2.2. Comparison between calcium carbonate (Calcite, Arg from cuttlefish bone, TArg from cuttlefish bone, and TArg-phytoplankton (A) Specific surface area (B) particle size.

Figure S2.3. Abrasion depth of TArg derived from cuttlefish bone, TArg derived from phytoplankton and Calcite slurries on Enamel, dentin and Calculus,

Fig.S.2.4.(A); FTIR of TArg after exposure to Brushite, (B); Corresponding subtraction of the FTIR spectra, (C) ); FTIR of TArg after exposure to dental calculus , (D); Corresponding subtraction of the FTIR spectra

1. **Materials and Methods**
   1. Oolitic Aragonite (Phytoplankton) as a second natural source of calcium carbonate

Synthetic calcite (CaCO_3_) was obtained from Sigma-Aldrich Chemical Company (St. Louis, MO, USA), while aragonite was obtained from phytoplankton (collected from the sand bank of the Bahamas). Aragonite minerals were cleaned to remove any remnant flesh and dried to a minimal water content of 3%. Dried aragonites were ground in an ultra-centrifugal type rotor mill and sieved through a sieve with a cut-off between 55 and 65 µm. To increase the reactivity of aragonite, the aragonite powder was treated with ammonium chloride (NH_4_Cl), at 20 ˚C in a solution of 55% water, 35% Arg powder, and 10% NH_4_Cl. The mixture was then centrifuged, sieved (opening of 55 µm), filtered, water washed until the pH was neutral and then dried in a tunnel drier.

1. **Supplementary Figures**


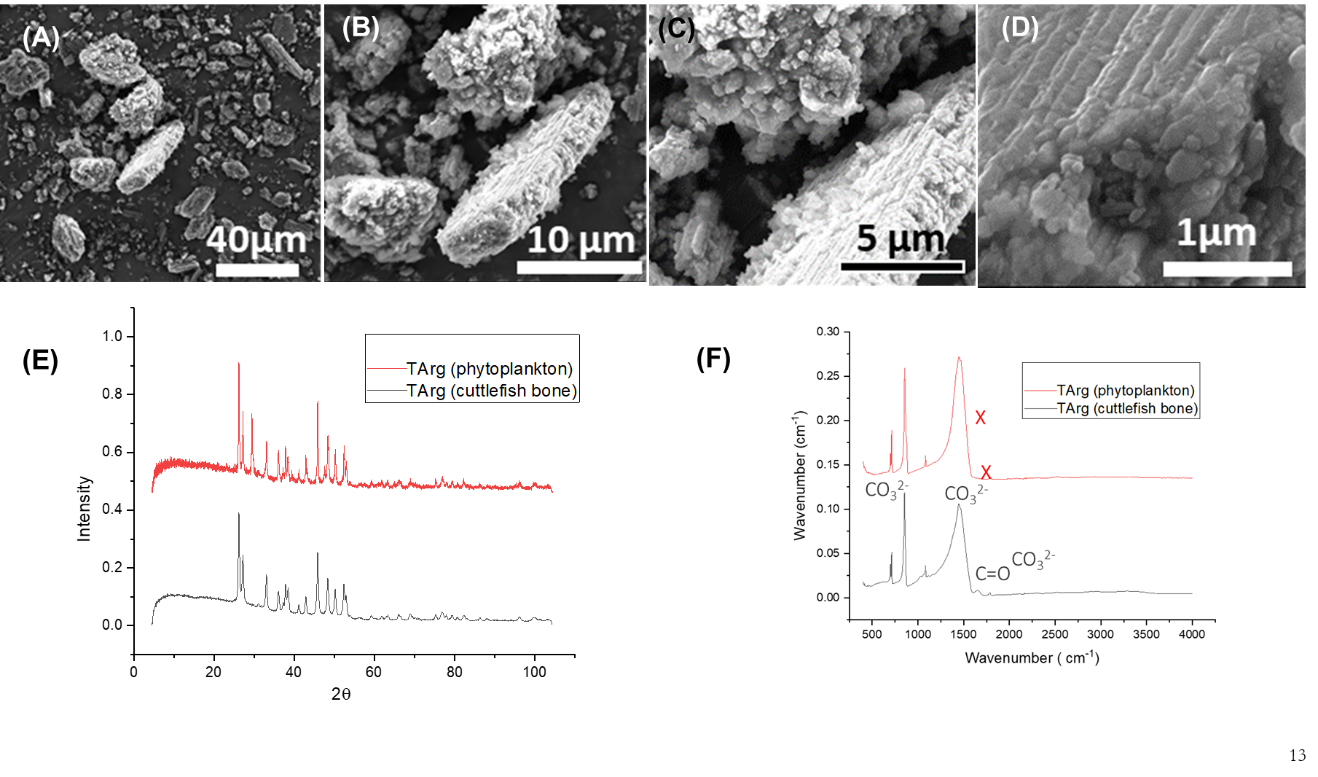


*Fig. S2.1. (A-D) SEM micrographs of the phytoplankton Aragonite, (E-F) XRD and FTIR of TArg obtained from two different sources; (****Black****) Cuttlefish bone and phytoplankton (Red)*


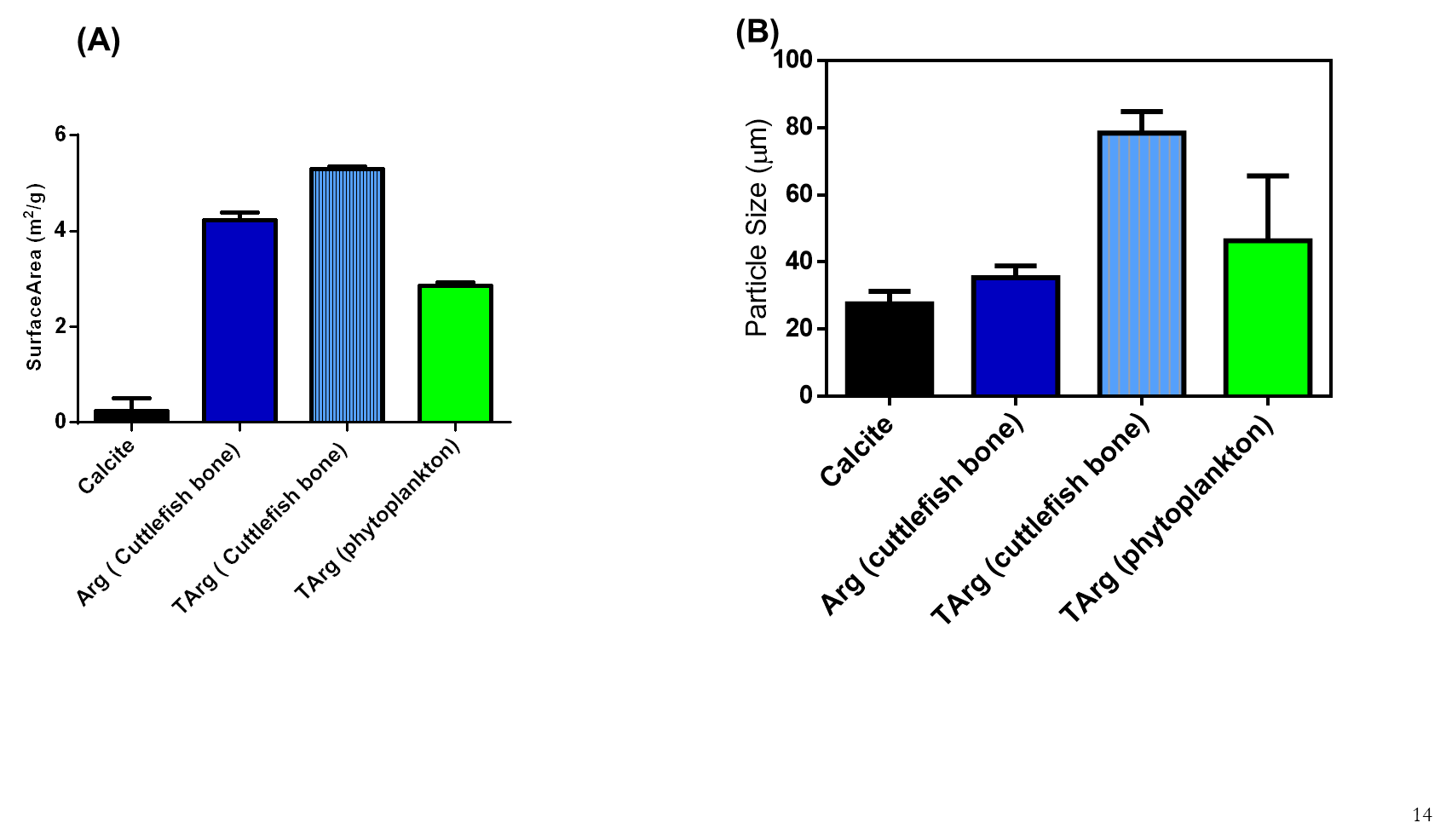


*Fig. S2.2. Comparison between calcium carbonate (Calcite, Arg from cuttlefish bone, TArg from cuttlefish bone, and TArg-phytoplankton (A) Specific surface area (B) particle size.*

*Fig.S.2.3. Abrasion depth of TArg derived from cuttlefish bone, TArg derived from phytoplankton and Calcite slurries on Enamel, dentin and Calculus,*

*
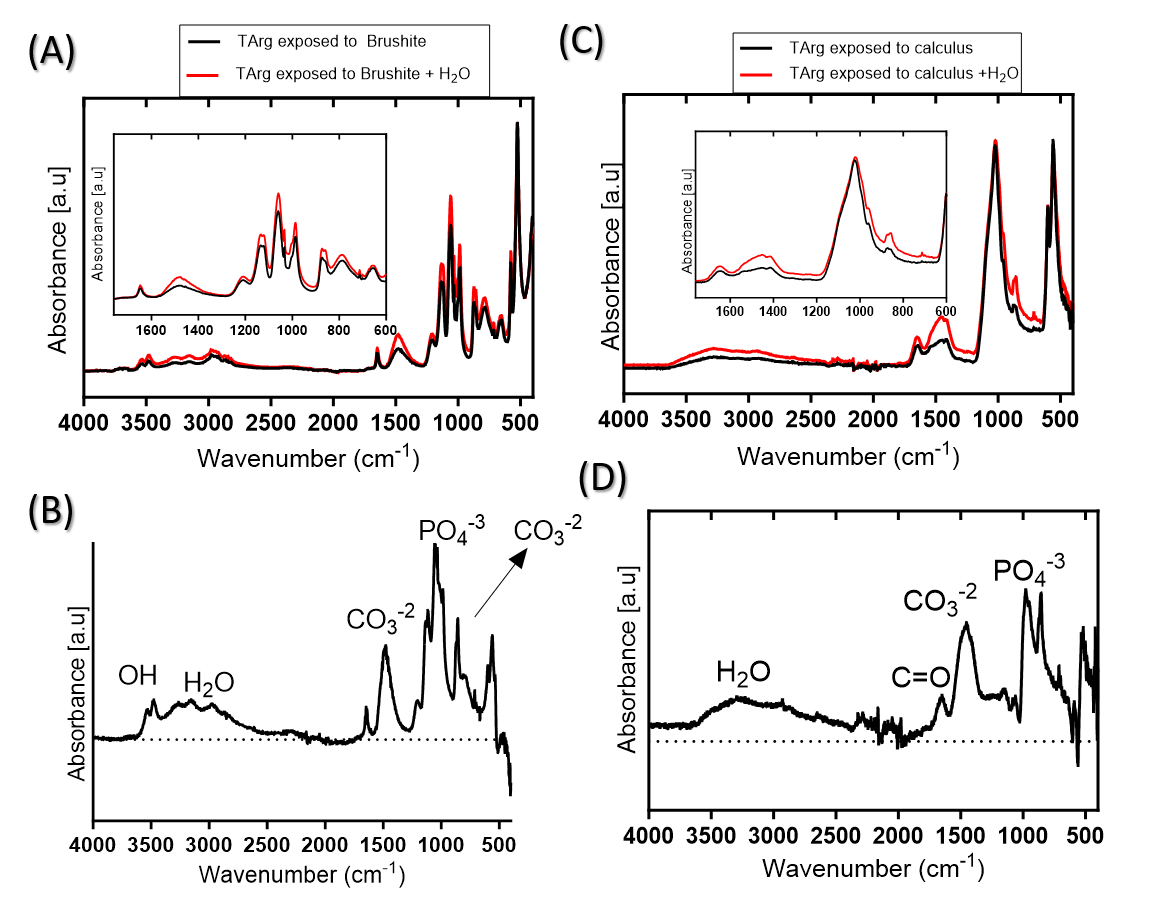
*

*Fig.S.2.4.(A); FTIR of TArg after exposure to Brushite, (B); Corresponding subtraction of the FTIR spectra, (C) ); FTIR of TArg after exposure to dental calculus , (D); Corresponding subtraction of the FTIR spectra*
